# Microbial and enzymatic biodegradation of aflatoxins and ochratoxins: mechanisms, applications, and emerging innovations

**DOI:** 10.1007/s00203-025-04683-8

**Published:** 2026-01-21

**Authors:** AO Aasa, SE Govender, S. Malgas, MS Thantsha

**Affiliations:** Department of Biochemistry, Genetics and Microbiology, University of Pretoria, Hatfield, 0028 South Africa

**Keywords:** Agricultural products, Aspergillus, Biocontrol, Food safety, Mycotoxin, Penicillium

## Abstract

Aflatoxins and ochratoxins are highly potent mycotoxins primarily produced by *Aspergillus* and *Penicillium* species, contaminating various agricultural commodities, especially cereals, nuts, and animal feeds. Chronic exposure to these mycotoxins is associated with liver cancer, immunosuppression, and developmental disorders, posing significant risks to public health and socioeconomic stability in numerous developing countries. Detoxification of mycotoxins has traditionally depended on physical and chemical methods, which exhibit limitations such as partial efficacy, nutrient loss, changes in food quality, high energy requirements, and environmental issues. Biological detoxification has recently garnered significant attention as a sustainable, safe, and eco-friendly alternative. This method utilises microorganisms, including bacteria, yeast, and fungi, along with their enzymes and metabolites, to transform mycotoxins into less toxic or non-toxic compounds, while maintaining the nutritional and sensory quality of food and feed. This review systematically analyses the recent advancements in the understanding of the microbiological and enzymatic mechanisms of aflatoxin (AFB) and ochratoxin (OTA) degradation. It emphasises the function of essential enzymes such as aldehyde dehydrogenase, amidohydrolase, carboxypeptidases, laccases, manganese peroxidases and oxidases, transforming AFB_1_ and OTA into less toxic compounds like AFD_1_, AFQ_1,_ L-β-phenylalanine and OTα. Industrial applications of these enzymes in feed and food processing are discussed. Contemporary challenges, including incomplete degradation, the formation of unknown by-products, and the variability of enzyme performance across different food matrices, are reviewed. The review proposes strategic approaches to enhance biological detoxification efficiency. These insights provide a framework for developing scalable, safe, and effective biotechnology solutions to mitigate mycotoxin contamination in the global food chain.

**Graphical abstract:**

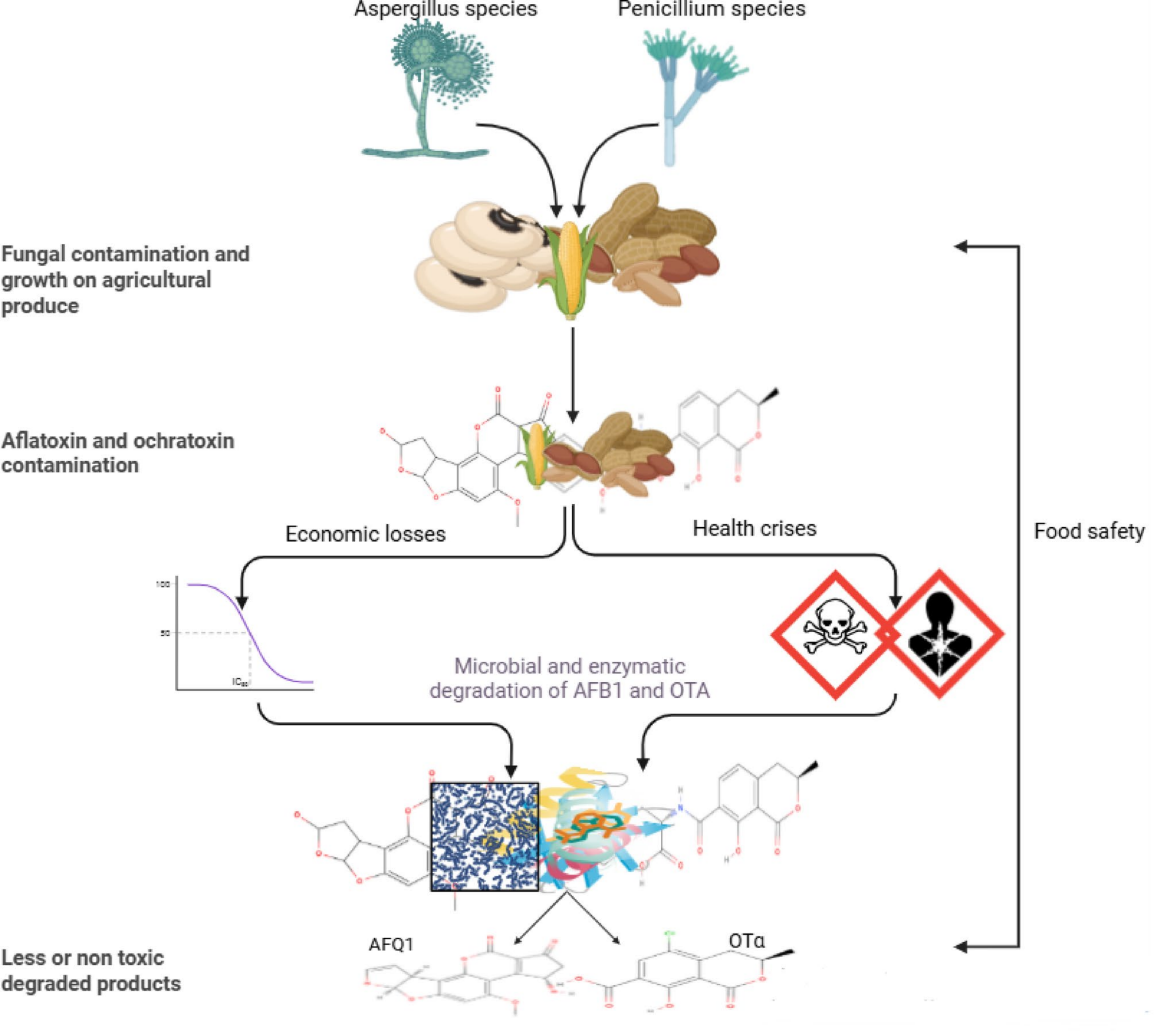

## Introduction

**Fig. 1:**
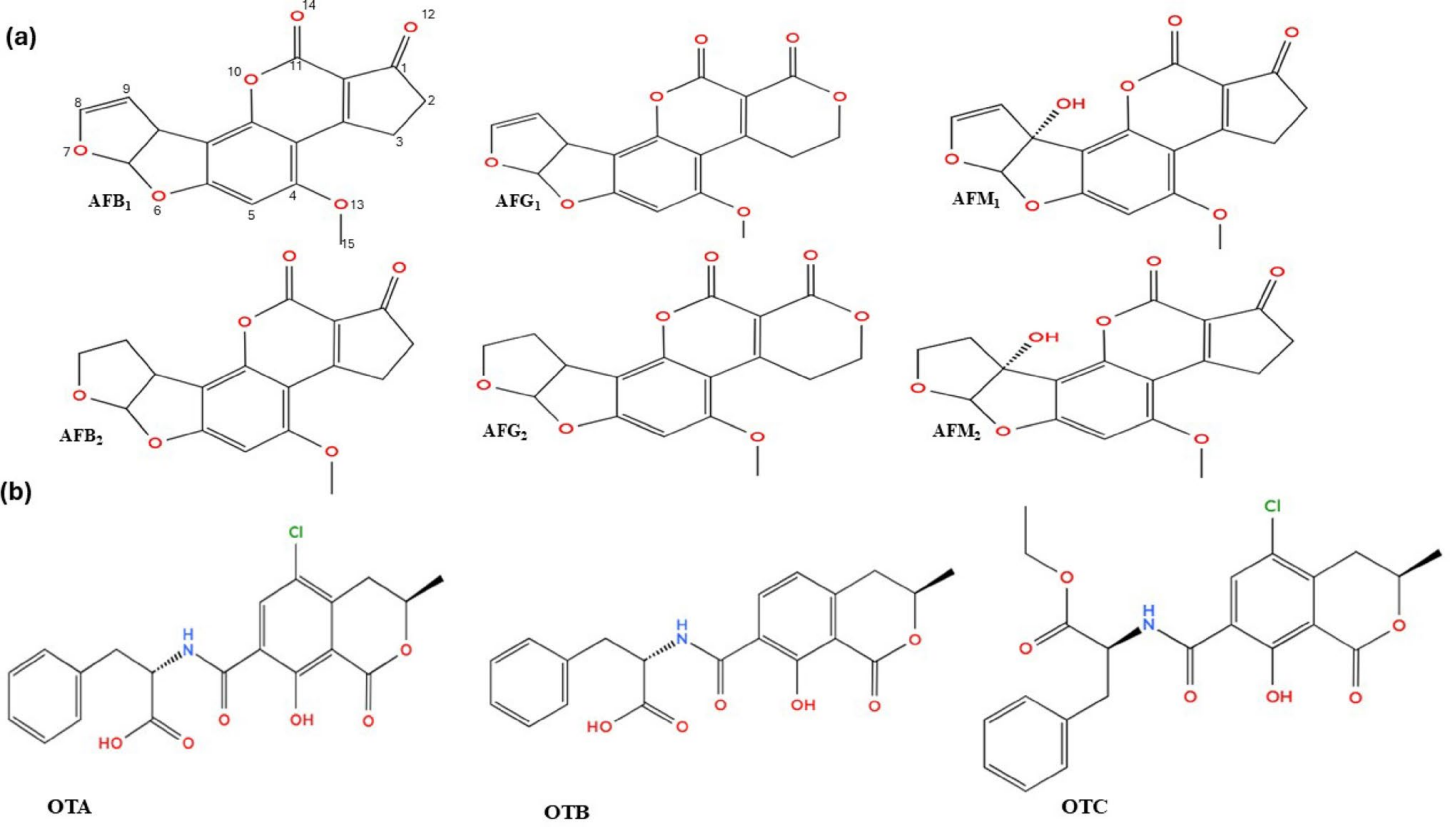
**a** Chemical structures of aflatoxin B_1_, B_2_, G_1_, G_2_, M_1_, and M_2_, and **b** of OTA, OTB and OTC

Mycotoxins, secondary metabolites of toxigenic fungi, present a significant concern in food products (Oliveira et al. 2014). Mycotoxins in food are produced by two groups of fungi, namely, field fungi, infecting crops before harvest, and storage fungi, which only appear after harvest (Tola et al. 2016). Aflatoxins (AFs; B_1_, B_2_, G_1_, G_2_, M_1_ and M_2_) and ochratoxins (A, B, and C), whose chemical structures are shown in Fig. 1a and b, are among the major mycotoxins. AFs are produced by toxic *Aspergillus* species, such as *A. flavus*,* A. nomius*, and *A. parasiticus*, among others, with *A. flavus* being the major producer. Globally, an estimated 5 billion people are exposed to AFs, making them the most concerning mycotoxins (Strosnider et al., 2006). Their presence in various food products has been frequently reported (Gbashi et al. 2020; Tebele et al. 2020). Aflatoxins can lead to widespread food insecurity by decreasing the value and quantity of agricultural products, which can have an impact on humanity on different levels including health, economic, political, and social (Pickova et al. 2021).

Four of the 18 identified AFs, aflatoxin B_1_ (AFB_1_), aflatoxin G_1_ (AFG_1_), aflatoxin B_2_ (AFB_2_), and aflatoxin G_2_ (AFG_2_), are widespread and can endanger human and animal health (Frisvad et al. 2019). The order of severity and toxicity of aflatoxins is AFB_1_˃AFG_1_˃AFB_2_˃AFG_2_ (Adebo et al. 2019). The lactone ring within the coumarin ring and the 8–9 double bond of the difuran ring are responsible for aflatoxin toxicity. A double bond at the 8–9 position in AFB_1_ and AFG_1_ makes them more potent than AFB_2_ and AFG_2_, which lack a double bond (Afshar et al. 2020). AFB_1_ exhibits toxicity at three primary sites: The first site is at positions 1,2,3 and 14 located at cyclopentenone ring, the second site at positions 10, 11, and 15 in the lactone ring and third site is the 8,9-double bond of the furan ring which interact with protein and nucleic acid causing carcinogenesis and gene mutation (Cheng et al. 2023). AFB_1_ can thus have major negative effects on human health and livestock productivity. It is widely regarded as the most potent liver carcinogen in humans and animals, classified as a group 1 “carcinogen to humans” by the International Agency for Research on Cancer (IARC). It is also capable of blocking RNA, DNA, and protein synthesis in humans (González-López et al. 2021). As a result, several African countries, including South Africa, have set AFB_1_ regulations in food, particularly cereals. In South Africa, AFB_1_ limits are set at 5 µg/kg for cereals, 0.05 µg/kg for milk and AFs limits of 10 µg/kg for all foods, including cereals (FAO, 2004; Chilaka et al. 2022). Some authors report even lower maximum limits for aflatoxins in cereals, such as 4 µg/kg (Gebremeskel et al. 2019).

On the other hand, ochratoxins are produced by various species of *Aspergillus* and *Penicillium*. They contaminate various agricultural products, including cereals, wine, coffee, and spices (Fazekas et al. 2002). There are three types of ochratoxins: ochratoxin A (OTA), ochratoxin B (OTB), and ochratoxin C (OTC). The most prevalent and toxic ochratoxin is OTA, which is produced by several *Aspergillus* and *Penicillium* species, including *A. carbonarius*,* A. ochraceus*,* P. nalgiovense*,* P. solitum*,* P. chrysogenum*, and *P. nordicum* (Masoud and Kaltoft 2006; Merla et al. 2018). Consumption of foods contaminated with these species has been linked to several diseases in both humans and animals (Otero et al. 2020). The National Food Safety Standard of China (GB2761-2017) sets OTA limits for grains, beans, wine, and coffee in the range of 2–10 µg/kg, while the Hygienic Standard for Feeds of China (GB13078-2017) has a limit of 100 µg/kg. The European Commission (EC/1881/2006) regulates the maximum levels of OTA in foodstuffs like baby food, wine, juice and coffee in the range of 0.5–10 µg/kg, with an average of 5 µg/kg for OTA in unprocessed cereals and 30 µg/kg in spices (FAO, 2004; Duarte et al. 2010). The European Commission (EC/576/2006) has established OTA limits for components in supplemental and compound feeds for pigs and poultry, ranging from 50 to 250 µg/kg (Agriopoulou et al. 2020). Nevertheless, many African countries, including South Africa, have no regulatory limitations on OTA (FAO, 2004).

Due to the negative impact of AFB_1_ and OTA on economic and human health, preventive strategies and degradation of these mycotoxins in food are necessary. Preventive measures like good agricultural practices, and detoxification can be achieved through physical, chemical, or biological treatments. The currently employed physical and chemical treatments of mycotoxins include among others, gamma and electron beam irradiation, activated carbon, cold plasma, electrolytes, montmorillonite, ultraviolet and pulsed light, metal-organic frameworks, and microwave heating (Ten Bosch et al. 2017; Liu et al. 2024; Akhtar et al. 2025). Physical and chemical methods have limitations such as loss of product quality may alter the taste and nutritional content of food, and they are also non-eco-friendly, costly, and may pose safety concerns (Söylemez and Yamaç 2021). Also, the use of chemical to increase agricultural productivity can damage life of living organisms, and their environment (Rasool et al. 2021).

Besides, the consumers demand safe, high-quality, minimally processed food with fewer chemical preservatives and additives (Nasrollahzadeh et al. 2022). Therefore, green technology, which includes the use of biological agents (microbes and enzymes) is used for food safety and security. The biodegradation techniques are efficient, cost-effective and eco-friendly (Saravanan et al. 2023). Several microbes including *Lactobacillus sp.*,* Agaricus campestris*,* A. niger*, and plant growth-promoting rhizobacteria (PGPR) such as *Bacillus*,* Enterobacter*,* Pseudomonas*, and *Rhizobium* sp., have been reported to have the ability to detoxify mycotoxins and improve food security (Fang et al. 2020; Söylemez et al. 2024; Thomas and Matthew, 2024). Integration of these microbes and their enzymes into food products not only detoxifies toxins but can enhance the function and structure of the microbiota (Jelku and Sangma 2024). Various enzyme-based biological techniques have been employed to detoxify the AF and OTA contamination (Loi et al., 2017). These enzymes include among others, oxidoreductases, hydrolases and peroxidases. The activities of these microbes and their enzymes depend on several aspects, such as the biological diversity (Shaji et al. 2024).

As a result, there is a high demand for research into effective, targeted, feasible, and environmentally biodegradable approaches. Recent efforts have focused on the biodegradation of mycotoxins using bacterial and fungal strains and their enzymes (Chen et al. 2018; Alimi et al. 2025). This review explores the principles and mechanisms of the enzymatic degradation of aflatoxins and ochratoxins in major food commodities. It also discusses their resultant degradation products and the potential industrial applications of the mycotoxin-degrading enzymes. It aims to improve our understanding of mycotoxin-degrading enzymes and offer theoretical and practical support for future research efforts on enhancing food safety. It highlights a multidisciplinary to utilise biological processes and advanced analytics for the creation of more sustainable mycotoxin-degrading microbes.

### Recent global contamination report of aflatoxins and ochratoxins in food matrices

The presence of aflatoxins and ochratoxins has been reported in food matrices worldwide. However, countries with humid and warmer climates shoulder a significant portion of the global aflatoxin problem (Jallow et al. 2021). In Ethiopia, aflatoxin contamination affects agricultural products like cottonseed, soybeans, peanuts, tree nuts and cereals like maize with a concentration of 864.66 µg/kg, which is far above the acceptable regulation of 20 µg/kg (Gelaye 2024). In Uganda, total aflatoxins were detected in maize, millet, sesame seeds and sorghum, ranging from 0 to 68.2 µg/kg, while OTA concentration ranged from 0.1 to 16.4 µg/kg. The mean concentration of aflatoxins detected was 11.8 µg/kg, exceeding the Ugandan national regulatory limits of 10 µg/kg in cereals (Echodu et al. 2019). Occurrence of aflatoxins and ochratoxin A was reported in seventy South African food spices, with the concentrations ranging from 3 to 19 µg/kg and 4–20 µg/kg for OTA (Motloung et al. 2018).

Aflatoxins and ochratoxins have been proven to be present in some food commodities in Europe. AFB_1_ and OTA were detected in the maize and wheat from different growing areas in Albania. The incidence rate of AFs in maize and wheat samples was 71 and 18% respectively. The concentration of AFs in maize exceeded maximum permitted levels in 36% of the samples, with the highest concentration of 2057, 2944 and 3550 µg/kg recorded, while 3 samples were reported to be contaminated with the OTA (Topi et al. 2023). The highest prevalence of AFs was detected in maize samples from Croatia (40%) and Serbia (84%) in the year 2021 due to an increase in temperature above 35 °C. In addition, many analysed samples harboured AFB_1_ at concentrations slightly higher than the ELISA’s limit of quantification (Pleadin et al. 2023). In Turkey, AFB_1_ was detected in chocolate wafer (8.7%), milk (19.6%), and bitter chocolate (13.3%) with concentration ranging from 0.15 to 2.04 µg/kg across all the samples while the OTA concentrations ranged from 0.18 to 0.75 µg/kg with incidences of 17.4% in chocolate wafers, 22.8% in milk chocolate and 46.7% in bitter chocolate (Kabak 2019).

In Asian countries, AFB_1_ and OTA were detected in food commodities. A dietary exposure to mycotoxins was conducted in three provinces in Northern Vietnam. The results reveal that all the analysed maize, peanut, rice and sesame were contaminated with mycotoxins, and AFB_1_ was the most frequently detected and widely distributed across the samples ( Do et al., 2020). Sixty-eight samples of dried chilli from Myanmar were screened for AFB_1_ and OTA contamination; 97% and 91% were contaminated with AFB_1_ and OTA, respectively. For AFB_1_, 37.9% exceeded Myanmar FDA’s maximum limit of 20 µg/kg, while 56.1% exceeded 10 µg/kg of the EU’s limit and 16.1% exceeded the EU’s limit of 20 µg/kg for OTA (San Phyo et al. 2024). High incidence of aflatoxins was detected in rice from China, with few samples exceeding the national limits of 10 µg/kg (Sun et al. 2017). Also, Aflatoxin was detected in Chinese spices, with the largest concentrations seen in chilli, pepper, and prickly ash (Zhao et al.,^2013^). Furthermore, 250 maize and rice bran samples from Cambodia, Myanmar, Laos and Thailand reported to be 100% contaminated with aflatoxins (Siri-anusornsak et al. 2022).

## Aflatoxins biodegradation

Microorganisms (fungi and bacteria) can degrade aflatoxins by producing substances that modify the structure of mycotoxins, transforming them into low- or non-toxic compounds (Guan et al. 2021). These microbial strains have been suggested for use as additives in animal feeds and oriental fermentation conditions, for mycotoxin degradation (Chlebicz and Slizewska, 2020). Reports on the fungi and bacteria degradation ability of AFB_1_ are discussed below.

### Fungi

Several types of filamentous fungi can degrade aflatoxins. Using high-performance liquid chromatography (HPLC), Chlebicz and Slizewska (2020) observed significant detoxification of AFB_1_by *Saccharomyces cerevisiae* after 6–24 h of incubation in phosphate-buffered saline. A salt-tolerant yeast, *Candida versatilis* CGMCC 3790, degraded 20 ng/mL AFB_1_ in a liquid medium, with degradation rates ranging from 41.23 to 100%. The four products identified were C_11_H_10_O_4_, C_13_H_12_O_2_, C_14_H_10_O_4_, and C_14_H_12_O_3_ (Li et al. 2018a). *Aspergillus terreus* strain HNGD-TM15, isolated from soil, efficiently degraded AFB_1_ in broth at a maximum degradation rate of 98.3%. A compound with molecular formula C_18_H_39_NO_3_ was detected to be a biodegradation by-product and tentatively named as 2-amino-1,3,34-octadecatriol. The identified biological mycotoxin degradation pathways include hydrolysis and lactone ring hydrogenation (Wu et al. 2023). *Aspergillus niger* ND-1 isolate removed 26.3% of AFB_1_ in nutrient broth within 48 h, while it removed 58.2% of AFB_1_ under optimal fermentation conditions within 24 h (Zhang et al. 2014). Also, *A. niger* RAF106 from tea degraded AFB_1_ within the ranges of 24, 48 and 72 h by 30.99, 52.71 and 88.59%. The degradation increased with prolonged incubation time, showing that *A. niger* degrades AFB_1_ in a time-dependent manner, producing end-products including C_16_H_34_O_9_, C_14_H_16_N_2_O_2_, C_14_H_16_N_2_O_3_, and C_23_H_48_O_8_ (Fang et al. 2020). *Trichoderma harzianum* AYM3 produced two metabolites, acetic acid and n-propyl acetate, which led to 78.8% degradation of AFB_1_ in coumarim medium. These metabolites were reportedly to be non-toxic to the HepaRG cell line and therefore are considered suitable for the application in the degradation of AFB_1_ (Madbouly et al. 2023). Notably, various similar metabolites are produced by other microorganisms, particularly bacteria, highlighting bacterial species as potential candidates for aflatoxin degradation.

### Bacteria

Various researchers have investigated the capability of different bacteria and/or their cell-free supernatants to degrade aflatoxin. Watanakij et al. (2020) used the extracellular fraction of *B. subtilis* BCC 42,005 to reduce AFB_1_in corn kernels by approximately 54% in 120 min, with optimal degradation obtained at 50 °C and pH 8.0. Reddy et al. (2010) reported that *Trichoderma viride*, reduced AFB_1_ in sorghum grains by 39%.

Clebicz and Slizewska (2020) recorded a 60% decrease in AFB_1_ concentration by *Lactobacillus* species. Wang et al. (2018) observed the biotransformation of AFB_1_ (C_17_H_12_O_6_) into C_12_H_14_O_4_ by *Bacillus licheniformis* (BL010) within 2 h. Ali et al. (2021) reported that ten bacterial isolates belonging to the genera *Enterococcus*,* Pseudomonas*, *Bacillus*, and *Stenotrophomonas* detoxified AFB_1_, AFB_2_, AFG_1_, and AFG_2_ in a liquid medium after incubation at 37 °C for 72 h. All isolates produced more than a 50% AFB_2_ and AFG_1_ reduction; six attained more than a 90% AFG_1_ reduction, while five exceeded 90% AFB_2_ reduction. *Pseudomonas fluorescence* SZI completely (99%) detoxified AFG_2_, AFB_2_, and AFB_1_. *Pseudomonas aeruginosa* degraded 99.6% AFB_1_ within 72 h in moldy maize, thereby reducing the AFB_1_ cytotoxicity and mutagenicity against the CCK-8 cell line (Xu et al. 2023). AFD_1,_ C_16_H_17_O_6,_ C_16_H_15_O_7,_ C_12_H_23_N_7_ O_2_ were detected as the biodegradation products of *Bacillus safensis* HHH526, and *Priesta megatarium* HBB522. Bacteria reduced the AFB_1_ and total AFs in dried figs by more than 50% while the limited degradation activity was observed in the yeast (Öztürk Köse and Biyik 2025). Probiotic bacteria from yoghurt showed the maximum 83.8% degradation of AFB_1_ at 30 °C after 48 h of incubation in Luria-Bertani (LB) medium. *B. cereus* XSWW9 degraded 86.1% of AFB_1_ after incubation at 37 °C for 72 h. It is hypothesized that the extracellular proteins produced by the bacteria are involved in the degradation process (Xue et al. 2023). The application of these bacteria and their enzymes for degradation of AFB1 may also improve the nutritional properties of the treated products. Hence, microbial enzymes involved in mycotoxin biodegradation are briefly discussed below.

### Enzymatic degradation of aflatoxins

Biodegradation of mycotoxin is a safe and eco-friendly approach that can reduce the concentration of aflatoxins in food commodities (Khan et al. 2020). A potential approach for the effective, specific, and environmentally friendly degradation of mycotoxins is enzymatic degradation, which is a component of biodegradation (Fang et al. 2023). Enzymatic recognition of aflatoxins, particularly aflatoxin B_1_, is influenced by the planar polyaromatic structure and reactive furan lactone rings of the toxins. Detoxifying enzymes bind aflatoxins via hydrophobic and π-π interactions, which accurately position the molecule within the active site. Hydrolytic enzymes facilitate the opening of lactone rings, disrupt molecular planarity, and eliminate DNA-binding capacity, whereas oxidoreductases alter conjugated systems (Ouyang et al., 2025; Hasan et al., 2025). The complete multi-step enzymatic transformation is crucial for preventing the formation of reactive epoxide intermediates and ensuring irreversible detoxification.

Several enzymes have been reported to degrade aflatoxins. Table 1 provides an overview of the instances in which microbial enzymes and toxins influenced degradation. These microbial enzymes exhibit diverse and effective mechanisms for the degradation of aflatoxins, particularly AFB_1_, which is a potent and widely distributed mycotoxin. These enzymes are derived from fungi, bacteria, and plants, each providing distinct catalytic mechanisms that transform AFB_1_ into less toxic or non-toxic metabolites. For instance, laccase and manganese peroxidase from *Trametes versicolour and Phanerochaete chrysosporium*, demonstrate significant specific activity in acidic environments, attaining degradation rates of up to 95% via ring-opening and oxidation mechanisms. Bacterial enzymes, such as aflatoxin-detoxifying enzyme (ADTZ) derived from *B. licheniformis*, function optimally at near-neutral pH and moderate temperatures, facilitating the reduction of AFB_1_ to less harmful hydroxy or dialdehyde derivatives. Plant-derived horseradish peroxidase and microbial tyrosinase significantly enhance detoxification capabilities through the production of hydroxylated or quinone products. The enzymes exhibit a range of specific activities, from 1 to 2 U/mg to 200 U/mg, indicating significant potential for biotechnological applications in food and feed safety. Their activities occur within the optimal pH ranges of 4.5 to 8.0 and temperatures of 25 to 50 °C, indicating adaptability to diverse industrial and environmental conditions. The most common enzymes reported in the degradation of aflatoxins are briefly discussed below.

#### Aflatoxin oxidase (EC 1.13.12.20)

Aflatoxin oxidase (AFO) was the first reported AFB_1_- degrading enzyme (Liu et al., 2024). This enzyme, previously known as aflatoxin-detoxizyme, was expressed from a Chinese edible and medicinal fungus, *Armillaria tabescens*. It is an intracellular proteolytic enzyme, with limited similarity to other oxidases, but exhibiting high structural similarity to dipeptidyl peptidase III (DPP III) (Xu et al. 2017). AFO selectively targets the furan or pyran structure on AFB_1_. Treatment of aflatoxin with purified AFO resulted in molecular polarity change and fluorescence retention, indicating the enzymatic cleavage of the bis-furan ring (Cao et al. 2011). AFO can oxidise the bisfuran ring and produce hydrogen peroxide (H_2_O_2_) simultaneously. The produced H₂O₂ with the active AFO may then react with the methoxy group or other oxygen-connected carbons and open the furofuran ring of oxidised AFB_1_ (Wu et al. 2015). When a purified AFO from *A. tabescens* was inoculated with AFB_1_ for 72 h at pH 6.0 and 30 °C it yielded 80% degradation of the AFB_1_ (Sinelnikov et al. 2023).

#### Laccases (EC 1.10.3.2)

Laccases are multi-copper oxidases, capable of degrading and converting a wide range of pollutants and toxins to less toxic and non-toxic products (Cabral Silva and Venâncio, 2021). They reduce molecular oxygen to water while catalysing the oxidation of phenolic and non-phenolic aromatic compounds (Jones and Solomon 2015). Laccases could degrade AFB_1_ by the addition of hydroxyl groups at carbon 11 and 10, resulting in an open ring, or acting on the terminal furan ring of AFB_1_ (Okwara et al. 2021). The most reported degraded products from laccase activity include epi-AFQ_1_, AFQ_1_, AFB_1_ aldehyde, AFB_1_-diol, AFB_2a_, and AFM_1_. Laccase (Lac2) extracted from white-rot fungi, *Cerrena unicolor* 6884, transforms AFB_1_ through 3-R-hydroxylation and produces a less toxic compound, aflatoxin Q_1_ (Zhou et al. 2020). A thermo-alkali-tolerant recombinant laccase form *B. swezeyi* (BswLac) transforms AFB_1_ within 24 h, producing compounds which could be referred to as AFB_1_−8,9-epoxide, AFQ_1_, AFM_1_ and epi-AFQ1 (Mwabulili et al. 2024). Lac-w from *Weizmannia coagulans* 36D1 catalyse the direct oxidation of AFB_1_ at 30 °C, pH 9.0, within 24 h, resulting in the degradation rate of 88% (Hao et al. 2023). A recombinant laccase (fmb-rL 103) from *Bacillus vallismortis* (fmb-103) degraded AFB_1_ with a degradation efficiency of 60% at pH 7.0 and 37 °C. Immobilised laccase (BF-NH_2_-Lac) demonstrated > 90% degradation of AFB_1_ in corn oil and buffer within 5 h. The AFB_1_ was transformed into non-toxic AFQ1without altering the quality of the oil (Rasheed et al. 2024). A relatively high concentration of 500 µg/kg of AFB_1_ was detoxified by 0.2 µM of a recombinant laccase (BsCotA; *B. subtilis*) at pH 7 and 37 °C. AFQ_1_ and epi-AFQ_1_ were identified as the degradation products through oxidation mechanisms (Subagia et al. 2024). However, for the most part, the mechanisms by which the enzymes catalyse the degradation remain unknown (Bian et al. 2022).

#### Lactonases (EC 3.1.1.81)

Acyl-homoserine lactone (AHL) lactonases are a group of bacterial metalloenzymes that degrade AHL and disrupt gram-negative quorum-sensing detection mechanisms. AHL is involved in biofilm formation and possesses a lactone ring in its structure (González Pereyra et al. 2019). Lactonases hydrolyse the ester bond of the lactone ring, rendering the signal molecule inactive (Sakr et al. 2013). It was postulated that lactonase and reductase produced atoxigenic *A. flavus* JZ2 and GZ15 were involved in the degradation of AFB_1_, and destroyed the furofuran and lactone rings, resulting in the formation of two by-products, identified as C_15_H_20_O_5_ and C_11_H_16_O_4_ (Xing et al. 2017). Lactonase (AttM) isolated from *Bacillus sp*. showed the optimal AFB_1_ degradation of 86.78% at pH 8.5, and 80 °C, within 24 h. It was observed that the AttM detoxifies the AFB_1_ by disrupting the endolipid structure of AFB_1_ producing AFD_1,_ which lack the lactone moiety, as a degradation by-product (Cheng et al. 2023).

#### Manganese peroxidase (EC 1.11.1.13)

Manganese peroxidase (MnP) belongs to the class heme peroxidase, catalysing hydrogen peroxide-dependent oxidation of (Mn^2+^) to manganese III (Mn^3+^) to form complexes with oxalic acid, malonic acid, and dicarboxylic acids (Lueangjaroenkit et al. 2019). The catalytic cycle of MnP starts with an indigenous ferric enzyme and H_2_O_2_ to generate compound I, called Fe^4+^ oxo porphyrin complex, while mono-chelated Mn^2+^ ions donate 1e^-^ to porphyrin, which leads to formation of compound II by donating 1e^-^ from Mn^2+^ to form Mn^3+^ (Chowdhary et al. 2019). Peroxidase can catalyse a wide range of reactions, such as cleavage of benzyl oxygen, carbon-carbon, bonds, epoxidation, hydroxylation, n-methylation, and ortho-demethylation therefore wide possible degraded products is possible (Wang et al. 2019). For AFB_1_ degradation, the catalytic reaction of MnP requires the involvement of dicarboxylic acid, Mn²⁺ and H₂O₂. This observation is validated by the results from the study by Xia et al. (2022), which showed MnP from recombinant *Kluyveromyces lactis* strains (pKLAC1- Phsmnp, pKLAC1-Plomnp and pKLAC1-Phcmnp) degraded AFB_1_ to AFB_1_- 8,9-dihydrodiol through free radicals formed by the interaction of oxidised Mn³⁺ with the dicarboxylic acid, which enhanced the redox potential for the degradation. The mechanism of AF degradation by MnP involves oxidation of the furan ring to an epoxy derivative. Mnp from *Phanerochaete sordida* YK-624, a white-rot fungus, oxidised the terminal furan ring of AFB_1_ to form AFB_1_−8–9-epoxide. However, oxidation of the double bond of the furan ring and release of the epoxide ring could be dangerous. Therefore, AFB_1_ was then hydrolysed to AFB_1_- 8,9-dihydrodiol in the presence of hydrogen peroxide, thereby eliminating its mutagenic activity (WangJianqiao et al. 2011). Also, manganese peroxidase from *Punctularia strigosozonata* (PsMnp) can facilitate the degradation of AFB_1_ through hydrophobic and polar interactions across several amino acid residues (Yang et al. 2025). Peroxidase (0.015 U/mL) supplemented with rice bran and soybean meal extracts degrades 78.2% AFB_1_ (5 ng/kg) within 24 h (Kerstner et al. 2024). A recombinant manganese peroxidase (*Il*-MnP1) and dye-decolourising peroxidase (*Il*-DyP4) from *Irpex lacteus* were used to degrade AFB_1_ at 25 °C and pH 6.5. The enzymes acted on various toxic sites of the AFB_1,_ resulting in the new five less toxic degrading products, identified as C_15_H_10_O_5_, C_15_H_10_O_6_, C_16_H_8_O_7_, C_16_H_10_O_7_, and C_16_H_10_O_8_ (Xu et al. 2025).

**Table 1:** Degradation of aflatoxins by microbial enzymes

Enzymes	Mycotoxins	Enzyme’s source	Enzyme specific activity (U/mg)	Degradation rate (%)	Optimum pH	Optimum temperature(ºC)	Degradation products	References
Laccase, and MnP	AFB_1_	*Pleurotus eryngii-*	57–90	8	25–37	Less toxic AFB_1_	Branà et al., 2020	
Multicopper oxidase (StMCO)	AFB_1_	*Streptomyces thermocarboxydus *0.2	NM	7	30	AFQ_1_	Qin et al., 2021	
Laccase (LAC3)	AFs	*Saccharomyces cerevisiae *	3	74.2–90.3	5.7	30	NM	Liu et al.,^2020^
*Myxobacteria* aflatoxin degradation enzyme (MADE)	AFB_1_	*Myxococcus fulvus*	100	71.9	6	35	Less toxic aflatoxin	Zhao et al., 2011
AFG_1_	*Myxococcus fulvus*	100	96.9	6	35	Less toxic aflatoxin		
AFM_1_	*Myxococcus fulvus*	100	95.8	6	35	Less toxic aflatoxin		
Porin and peroxiredoxin	AFB_1_	* -*	NM	91.12 - 100	6 −10	60 - 80	AFD_1_	Adegoke et al., 2023
MnP – Mn peroxidase	AFB_1_	*Pleurotus ostreatus *	78	90	4–5	25	Less toxic aflatoxins	Yehia 2014
Laccase	AFB_1_	*Saccharomyces cerevisiae *	2	91	5.7	34	C_16_H_12_O_7_, C_14_H_16_N_2_O_2_, C_7_H_12_N_6_O_3_, C_24_H_30_O_6_.	Liu et al., 2021
Laccase	AFB_1_	*Trametes versicolor*	30	27–86	4.5	35	Non-mutagenic compound	Zeinvand-Lorestani et al., 2015
Lac+ ABTS+ AS+ SA	AFB_1_&AFM_1_	*Pleurotus pulmonarius -*		90	5	25	NM	Loi et al., 2016
Ery 4 laccase+ Syringyl-type phenols	AFB_1_	*Saccharomyces cerevisiae*	5	48–73	5	25	NM	Loi et al., 2018
Laccase	AFB_1_	*Trametes versicolor*	12–25	60–90	5.0	30 45	AFQ1-like derivatives, epoxide-opened products	Alberts et al., 2009
Manganese Peroxidase (MnP)	AFB_1_	*Phanerochaete chrysisporium*	20–30	70–90	4.5–5.0	30–37	Reduced furofuran ring products3	Wang et al., 2011
Peroxidase (HRP)	AFB_1_	Horseradish (*Armoracia rusticana*)	200–300	55–80	6.0–7.0	25 37	Hydroxylated AFB1	Loi et al., 2018
ADH (Aldehyde Dehydrogenase)	AFB_1_- dialdehyde	*Pseudomonas *	NA	50–60	6.0–7.0	30–37	Detoxified carboxylic acid derivatives	Mizuno et al., 2015

Where*AFs:* aflatoxins (B1, B2, G1, and G2); *AFB1:* aflatoxin B1, *MnP:*Manganese peroxidase,*Lac:* Laccase; *ABTS:2*2′-azino-bis-[3-ethylbenzothiazoline-6-sulfonic acid])*AS:* acetosyringone *SA:* syringaldehyde. *NM:* Not mentioned.

## Biodegradation of OTA

Given the benefits of improved safety, availability, flavour, and nutritional quality, many scientists have focused on using biological methods for the degradation or adsorption of OTA. Currently, various microorganisms, such as yeast, bacteria, and fungi, along with their enzymes, have been used for OTA elimination (Du et al. 2021). Some information on the fungal and bacterial degradation of OTA is highlighted below.

### Fungi and bacteria degradation of OTA

Some fungi play a crucial role in degrading OTA into less toxic or non-toxic compounds. Various non-toxigenic strains of fungi, including *A. niger*, *A. tubingensis*,* Rhizopus*, and yeast, have shown the ability to degrade OTA. *Aspergillus* sp. OA3 from Fu brick tea degraded 79.23% of OTA in culture medium over 7 days and maintained a high, stable degradation efficiency across 25–55 °C (Han et al. 2025). Rodríguez et al. (2024) isolated *Rhodosporidiobolus ruineniae* and *Meyerozyma caribbica* from the Ivorian robusta coffee. *R. ruineniae* showed adhesion to the hyphae and OTA adsorption, while *M. caribbica* degraded 50% of OTA in yeast extract and glucose liquid medium (YEG) at 25 °C after 24 h of incubation. *Yarrowia lipolytica* led to 50% degradation of OTA in grapefruits after 24 h of incubation, reducing decay and storage quality issues without any negative effects (Yang et al. 2015). *Agaricus campestris* OBCC 5048 degraded 46.67, 56–63.20% OTA, while *Lentinus strigosus* OBCC 5004 degraded 66.7–67.0% AFB_1_ at 28 °C within 1 h (Söylemez and Yamaç et al., 2021; Söylemez et al. 2024).

For bacterial degradation, various studies have been conducted on various bacterial species to investigate their OTA-degrading capacity. A significant reduction of OTA in stored wheat was achieved by *L. plantarum* (> 97%) and *L. graminis* (> 97%), followed by *P. pentosaceus* (> 81.5%) (Belkacem-Hanfi et al. 2014). The *Brevundimonas naejangsanensis* strain from soil completely degraded OTA and OTB into OTα and OTβ, respectively, within 24 h of incubation (Peng et al. 2022). *Lactiplantibacillus plantarum* detoxified OTA in a liquid medium at pH 3.0 (Møller et al. 2021). Chang et al. (2015) reported that cell-free extracts of *B. amyloliquefaciens* ASAG1 degraded 98.5% of OTA after 24 h of incubation, with no detectable OTA after 72 h. Two neutralisation pathways were detected: adsorption to the cell wall and degradation of the active site by the strain.

Rodriguez et al. (2011) studied the OTA degradation capabilities of *Brevibacterium* spp., showing complete (100%) degradation of OTA into OTα. Zhang et al. (2017) identified *Alcaligenes faecalis* as an OTA degradant due to its production of hydrolytic enzymes. Their study revealed that *(A) faecalis* minimised OTA exposure risk by degrading it into non-toxic OTα. In another study, *(B) velezensis* E2 removed 96.1% of OTA in a liquid medium after 48 h. The results suggested the possibility of alkaline hydrolysis to be the main mechanism, with ring-opened ochratoxin α (OP-OTα) detected as a by-product (Zhang et al. 2022). Additionally, Campos-Avelar et al. (2020) tested the OTA-degrading capabilities of sixty *Actinobacteria* strains in liquid and solid media. Thirty-three strains degraded OTA in liquid medium, while only five strains degraded it in solid medium. There is no mention of the common by-products, suggesting that Actinobacteria sp employed a different mechanism leading to generation of unknown by-products, possibly as a result of the synthesis of distinct metabolites or enzymes.

### Enzymatic degradation of OTA

Enzymatic recognition of ochratoxin A is primarily governed by its amide bond linking the isocoumarin moiety to phenylalanine, which is central to its toxicity. Detoxifying enzymes, particularly carboxypeptidases and proteases, recognize this linkage through active-site complementarity and hydrogen bonding, yielding ochratoxin α and phenylalanine, disrupting protein synthesis and significantly reducing nephrotoxicity (Ouyang et al., 2025; Liu et al. 2023).

The enzymes with proven OTA degrading ability are carboxypeptidase, amidohydrolase, proteases and peroxidase as summarised in Table 2. Many peptidases can hydrolyse OTA, exerting their detoxifying effect via cleavage of the amide bond between iso-coumarin and the L-phenylalanine residue. Furthermore, an enzyme specific for OTA degradation (ochratoxinase, OTase) obtained from *A. niger* W-35 and subsequently expressed in *Escherichia coli* BL21 degraded OTA at 85.1% for 12 h (Zhao et al. 2019). Crude enzymes from *A. tubingensis* M036 and M074 achieved OTA biodegradation of 97.5% and 91.3% at pH 5, and 80.3% and 75.3% at pH 7, respectively, after 24 h in a buffer solution containing OTA (40 ng/ml). Furthermore, the OTA-biodegrading fungi showed no OTA synthesis activity (Cho et al. 2016).

Furthermore, Table 2 presents microbial enzymes that degrade Ochratoxin A (OTA) and constitute a diverse and effective group of biological catalysts, demonstrating substantial potential for detoxification in food, feed, and environmental systems. These enzymes are derived from fungi, bacteria, and yeasts, differing in specificity, catalytic efficiency, and optimal operating conditions. The most effective detoxifiers are carboxypeptidase A from *A. niger* and ochratoxinase from *A. carbonarius*, both demonstrating degradation rates of 90 to 100% by hydrolysing OTA into the non-toxic metabolite ochratoxin α (OTα). Other fungal enzymes, including carboxypeptidase Y from *Saccharomyces cerevisiae* and peptidase A from *Penicillium chrysogeum*, demonstrate moderate to high activity, degrading 50 to 85% of OTA under mildly acidic conditions. Bacterial enzymes are crucial, with amidase from *Aminobacter* species demonstrating nearly complete detoxification efficiency at neutral to slightly alkaline pH, converting OTA to OTα and L-β-phenylalanine. Protease-type enzymes derived from *Bacillus amyloliquefaciens* and *Bacillus subtilis* exhibit the capacity to degrade 50 to 80% of ochratoxin A (OTA), generally under neutral pH conditions and moderate temperature ranges. Oxidative enzymes, including laccase from *Trametes versicolour*, facilitate OTA transformations via oxidation pathways, albeit with reduced efficiency and the production of more complex degradation products, such as quinone derivatives. In these enzyme systems, specific activities vary from 2 to 40 U/mg in fungal carboxypeptidases, with optimal pH values typically between 5.0 and 7.0, and temperature ranges from 28 to 40 °C. The primary detoxification mechanism entails the hydrolysis of the amide bond connecting the isocoumarin moiety of OTA to phenylalanine, resulting in OTα. These microbial enzymes collectively exhibit significant potential for integration into biotechnology-based strategies for mycotoxin control. The enzymes with proven OTA degrading ability are briefly discussed below.

#### Amidohydrolase (EC 3.5.1-2.1.1.1)

The amidohydrolases superfamily comprises a remarkable set of metal-dependent hydrolases, which catalyse the hydrolysis of a wide range of substrate-bearing amides or ester functional groups at carbon and phosphorus centres (Jayaraman et al. 2016). Specifically for OTA amidohydrolase activity is executed through the cleavage of the C-N bond or amide bond. The isoenzymes of N-acyl-l-amino acid amidohydrolase (NA) and amidohydrolase ADH3 from *Stenotrophomonas* sp. CW117 hydrolysed OTA through amide bond cleavage to produce OTα (Chen et al. 2022). In another study, 1 µg/kg of an amidohydrolase (ADH2) from *Lysobacter sp*. CW239 hydrolysed 50 µg/kg of OTA into the same products in 5 min (Fu et al. 2024).

A recently discovered amidohydrolase, PwADH, was shown to be highly effective in degrading OTA, attaining maximum activity at 45 °C and pH 7.5, without the addition of extra metal ions (Hu et al. 2024). The crude protein extracts of *Metarhizium robertsii* and *Metarhizium brunneum* significantly detoxified OTA and OTB, resulting in the non-toxic OTα and OTβ as the end products. Amidohydrolase MbAmh1 was identified as the main enzyme responsible for the degradation process. The enzyme has shown significant temperature adaptability, ranging from 30 to 70 °C, and pH stability from 4.0 to 7.0 (Wang et al. 2024). Evaluation of the OTA degradation capabilities of three amidohydrolases (ADH1, ADH3, and AMD3) extracted from *P. pastoris* showed OTα and L-β phenylalanine as their degradation products. About 1 µg/kg of crude ADH1 completely degraded 50 µg/kg of OTA in 14 min, while the purified enzyme took 30 min. At the same concentrations, crude AMD3 and ADH3 degraded OTA in 9 and 24 min, respectively, while their purified enzymes did so in 24 and 42 min (Zhang et al. 2024). A novel amidohydrolase from *Acinetobacter baumannii* achieved 93% of OTA degradation within 5 min and degraded 87% of OTA in grape juice within 3 at 20° C, while retaining the quality of juice. The enzyme cleaves the amide bond of the OTA, producing OTα as the less toxic by-product (Guo et al. 2024).

#### Carboxypeptidase (EC 3.4.16–18)

Carboxypeptidase is a zinc-containing metallocarboxypeptidase belonging to the family of M14 (CPA/B). Carboxypeptidases hydrolyse the C-terminal peptide bonds of proteins or polypeptides, releasing free amino acids (Song et al. 2021). They exhibit exopeptidase and cleave the C-terminal amino acid of peptides during their catalytic activity (Ábrahám et al. 2025). Serine-type-D-ala-D-ala and D-ala-D-ala-carboxypeptidase from microbes are involved in the breakage of the OTA peptide bond. CPA can hydrolyse the amide bond of OTA to produce OTα and L-phenylalanine, making it suitable for OTA degradation in feed and food (Ming et al. 2023).

Xiong et al. (2020) engineered *Pichia pastoris* to express a mature CPA (M-CPA) with the ability to degrade up to 93.36% of OTA at the optimum pH and temperature of 8 and 40 °C. A decrease in OTA concentration and its biotransformation into OTα by *Brevibacterium* sp. strain was linked to its production of carboxypeptidase (Rodriguez et al., 2011). A carboxypeptidase from *Acinetobacter* sp. hydrolysed OTA to OTα and L-β phenylalanine (Sánchez-Arroyo et al. 2024).

#### Proteases (EC 3.4.21–99.21)

Proteases are enzymes that completely or partially break down proteins into smaller peptides. Proteases differ in their active sites, substrate specificity, and catalytic mechanism (Kumari et al. 2015). Proteases also hydrolyse the amide bond of OTA. Several microorganisms, including *Lactobacillus* and *Bacillus* species, are reported to produce mycotoxin-degrading proteases (Aguirre et al. 2014; Yu et al. 2019). Metallopeptidase with amidohydrolase from *Alcaligenes faecalis* HNGD-HTN06, degraded 98% of OTA, and hydrolysed the amide bond into ochratoxin α at pH 8 and 37 °C, within 72 h (Hou et al. 2025). Purified protease from *Lactobacillus* sp. at 200 U/mL degraded 82.6% OTA after 18 h of incubation (Kholif et al. 2022). *Ananas comosus* bromelain cysteine-protease and bovine trypsin serine-protease were used for OTA degradation. The inactivation mechanism in T-2 by trypsin protease triggers the catalytic action of Ser195 and attacks the carbonyl of the amide bond in OTA, also Cys26 in bromelain protease attacks the carbonyl of the amide bond, and the amino group in amide interacts with Hys158, then breaks the amide bond, resulting in OTα and β-phenylalanine as by-products (Orozco-Cortés et al. 2023).

**Table 2:** Degradation of Ochratoxin A by microbial enzymes

Enzymes	Mycotoxin	Enzyme source		Degradation rate (%)	Optimum pH for degradation	Optimum temperature(ºC)	Degradation product(s)	References				
Enzyme mixtures (peroxidase, hydrolase)	OTA	*Trichoderma afroharzianum T22*		31.0–46.0	NM	37.0	NM	Dini et al. 2022				
Carboxypeptidase	OTA	*Bacillus amylolique-faciens ASAG1*		41.0–72.0	7.0	37.0	OTα	Chang et al. 2015				
Carboxypeptidase A(CPA)	OTA	*Brevibacterium* spp. 100.0			NM	30.0	OTα, L-β-phenylalanine	Rodriguez et al. 2011				
Carboxypeptidase	OTA	*Acinetobacter sp. Neg1 strain*		70.0	NM	28.0	OTα	Liuzzi et al. 2017				
Carboxypeptidase OTA *Bacillus subtilis* CW14				71.3	7.0	37.0	OTα	Xu et al. 2021				
Ochratoxinase OTA			*Aspergillus niger*	50.0	5.6–6.0.6.0	66.0	NM	Dobritzsch et al. 2014				
		*Pediococcus parvulus*		85.1	-	-	-					
		*Yersinia lipolytica Y-2*										
Peroxidase (POD)	OTA	*Armoracia rusticana*		27.0	7.0	30.0	Less toxic OTA	De Oliveira Garcia et al. 2022				
Carboxypeptidase	OTA	*Lysobacter sp. CW* 239 86.2 7.0				37	OTα	Wei et al. 2020				
N-acyl-L-amino acid amidohydrolase (AfOTase)	OTA	*Alcaligenes faecalis*			6.5	50.0	OTα	Zhang et al. 2019				
Recombinant Nudix hydrolase Nh-9	OTA	*Bacillus velezensis IS-6*		68.0	7.0	37	OTα	Jahan et al. 2023				
Lipase	OTA	*Aspergillus niger*		10–18	5.0	30	OTα	Farbo et al., 2018				
Amidase	OTA	*Aminobacter sp.*		⁓2	7.0–8.0	30–37	Otα + L-β-Phenylalanine	De Bellis et al., 2015				
Ochratoxinase (OTase)	OTA	*Aspergillus carbonarius*		3–7	6.0	30–37	Otα	Dobritzsch et al. 2014				
Laccase	OTA	*Trametes versicolor*		10–22	4.0–5.0	30–40	Chlorinated quinones, oxidised OTA derivatives	Prado et al., 2013				
Carboxypeptidase B-like enzyme	OTA	*Bacillus subtilis*		⁓6	7.0	37	Otα	Yu et al., 2017				
Peroxidase (PoDyP4)	OTA/OTB	*Pleurotus ostreatus*		83.0/98.3	5.0	20–40	6-OH-OTA/OTB-quinone	Xia et al. 2025				

Where: *OTA:* Ochratoxin A, *OTα* : ochratoxin α, *NM* : Not mentioned

## Aflatoxins and Ochratoxin A degradation pathways

The primary mechanism for detoxifying mycotoxins involves the capacity of microbes and their enzymes to alter, modify, or disrupt the C8-C9 double bond within the furofuran ring, lactone ring, cyclopentanone, and methoxy group of these compounds (Liu et al. 2019).

### Aflatoxin degradation reaction mechanisms and pathways

Aflatoxins can be degraded via various pathways, including oxidation, hydrolysis, hydroxylation, reduction of keto groups, loss of oxygen, and demethylation (Lu et al. 2023), with hydrolysis and oxidation being the most well-established methods. AFB_1_ has been recognised as the most toxic mycotoxin, has been the primary subject of research concerning mycotoxin degradation.

#### Oxidation

**Fig. 2:**
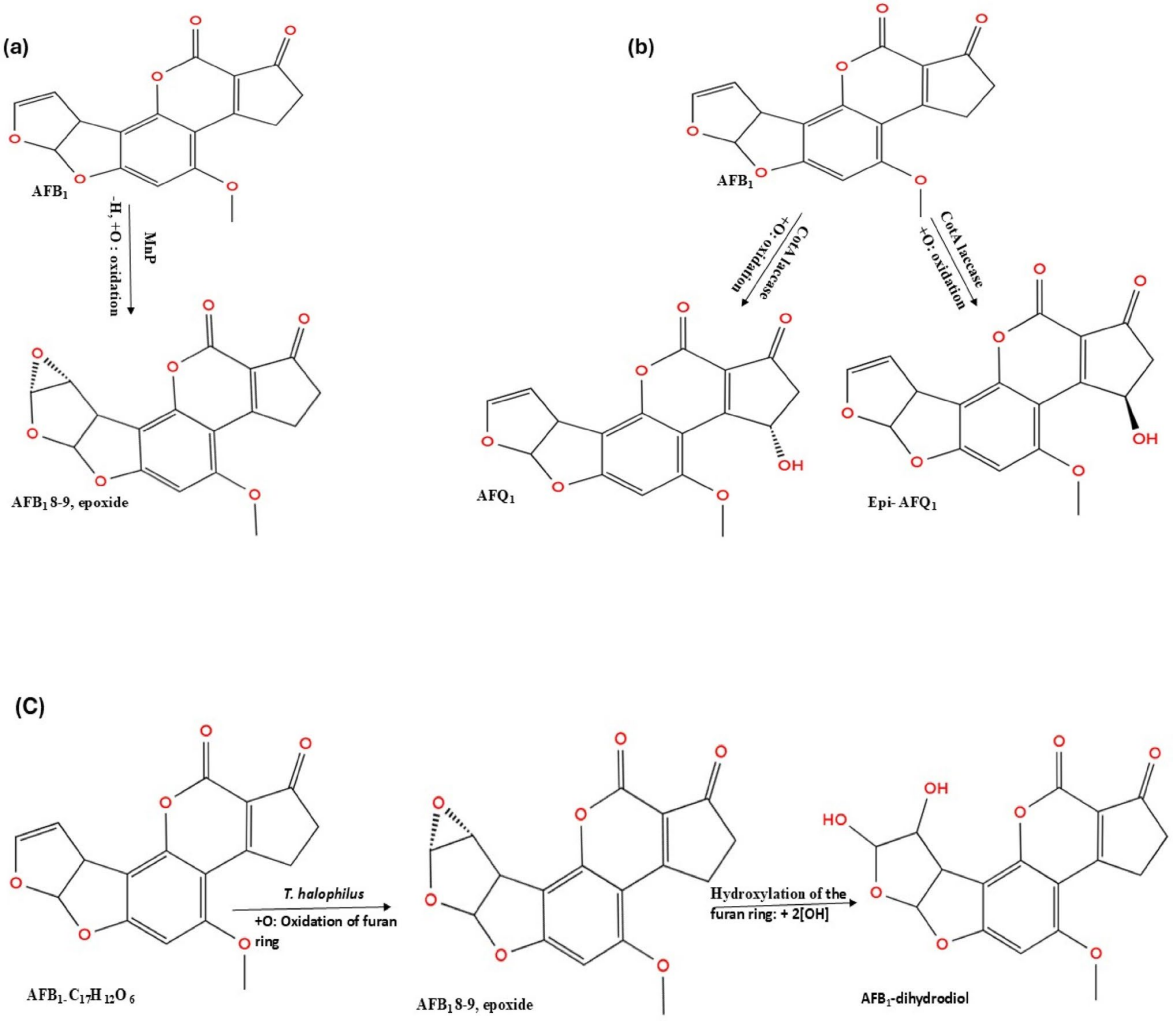
Oxidation pathway of Aflatoxin B_1_ biodegradation: **a** Oxidation by Manganese peroxidase from *Irpex lacteus*, the 8,9-vinyl bond of AFB_1_ was oxidised and oxygen atom was added to form AFB_1_- 8,9- epoxide, **b** Oxidation by laccase CotA from *B. subtilis*, attaches hydroxyl group to the AFB_1_ to form epi-AFQ_1_ and AFQ_1_ and **c** Oxidation by a novel aflatoxin oxidase (CotA laccase) from *T. halophilus* react with 8,9-difuran ring to form AFB_1_−8,9-epoxide which then converted to AFB_1_-dihydrodiol

The dihydrofuran ring containing an 8–9 double bond in AFB_1_ serves as a significant site for oxidation reactions due to the conjugation of the double bond with an oxygen atom (Arimboor 2024). AFB_1_ can undergo oxidation at the 4–5 double bond to yield an epoxide; however, it may also be converted to a less toxic compound via the cleavage and degradation of the furan and lactone rings. AFB_1_ undergoes oxidation to form an epoxide intermediate, AFB_1_−8,9-epoxide, facilitated by manganese peroxidase. During this reaction, the 8–9 bond of AFB_1_ undergoes oxidation by MnP via the addition of oxygen to the molecule (Fig. 2a), resulting in the transformation of AFB_1_ into AFB_1_−8–9-epoxide, which has a higher molecular weight (16 kDa) than AFB_1_ but is a less toxic compound (Wang et al. 2019). *Armillaria tabescens*, along with the aflatoxin oxidase it produces, is capable of disrupting the ether bond of the furan ring, thereby converting AFB_1_ directly to AFB_1_- 8–9 epoxide (Tomin and Tomić 2019). The oxidative degradation of AFB_1_ involves the addition of oxygen to the furan and lactone rings by CotA laccase, resulting in the formation of degradation products: AFB_1_-diol, epi-AFQ_1_, and AFQ_1_, as illustrated in Fig. 2b. AFB_1_-diol exhibits reduced toxicity compared to AFB_1_ due to the elimination of the double bond in the furan ring, which underlies its carcinogenic and toxic properties (Sun et al. 2023). The application of aflatoxin-degrading enzymes in food indicates that rCotA can facilitate the direct oxidation of AFB_1_ by utilising molecular oxygen as the final electron acceptor (Guo et al. 2021). Li et al. (2023) demonstrated that *T. halophilus* degrades AFB_1_ through an oxidation reaction, characterised by the incorporation of oxygen atoms into the furan ring double bond of AFB_1_. This process results in the elimination of - H groups, alongside the addition of oxygen, yielding AFB_1_−8,9- epoxide, then attaches hydroxyl group to oxidised AFB_1_ to form AFB_1_-dihydrodiol as products (Fig. 2c). The double bond oxidised by the *T. halophilus* is the oncogenic site of AFB_1_ used for the inhibition of RNA synthesis, therefore its disruption eliminates the carcinogenic activity of AFB_1_.

A novel aflatoxin oxidase, CotA laccase, derived from *B. licheniformis* transforms AFB_1_ into epi-AFQ_1_ and AFQ_1_, both of which are non-toxic to human cells. The enzymes modify AFB_1_ by adding a hydroxyl group to the C3 position, resulting in two products classified as epimers due to their similar spectra and identical characteristics. It is hypothesised that the hydrogen bonding and the van der Waals interactions play an important role in stabilizing CotA-AFB_1_, thereby facilitating the degradation of AFB_1_ (Guo et al. 2020).

#### Hydrolysis

The aflatoxin biodegradation process also involves the opening of a lactone ring through a hydrolysis reaction. In a study by Afsharmanesh et al. (2018), BacC oxidoreductase from *B. subtilis* UTB1 ruptured and hydrolysed the lactone ring of AFB_1_ to AFD_1_, followed by decarboxylation to AFD_2_ through breakage of the bond with the cyclopentenone ring.

**Fig. 3:**
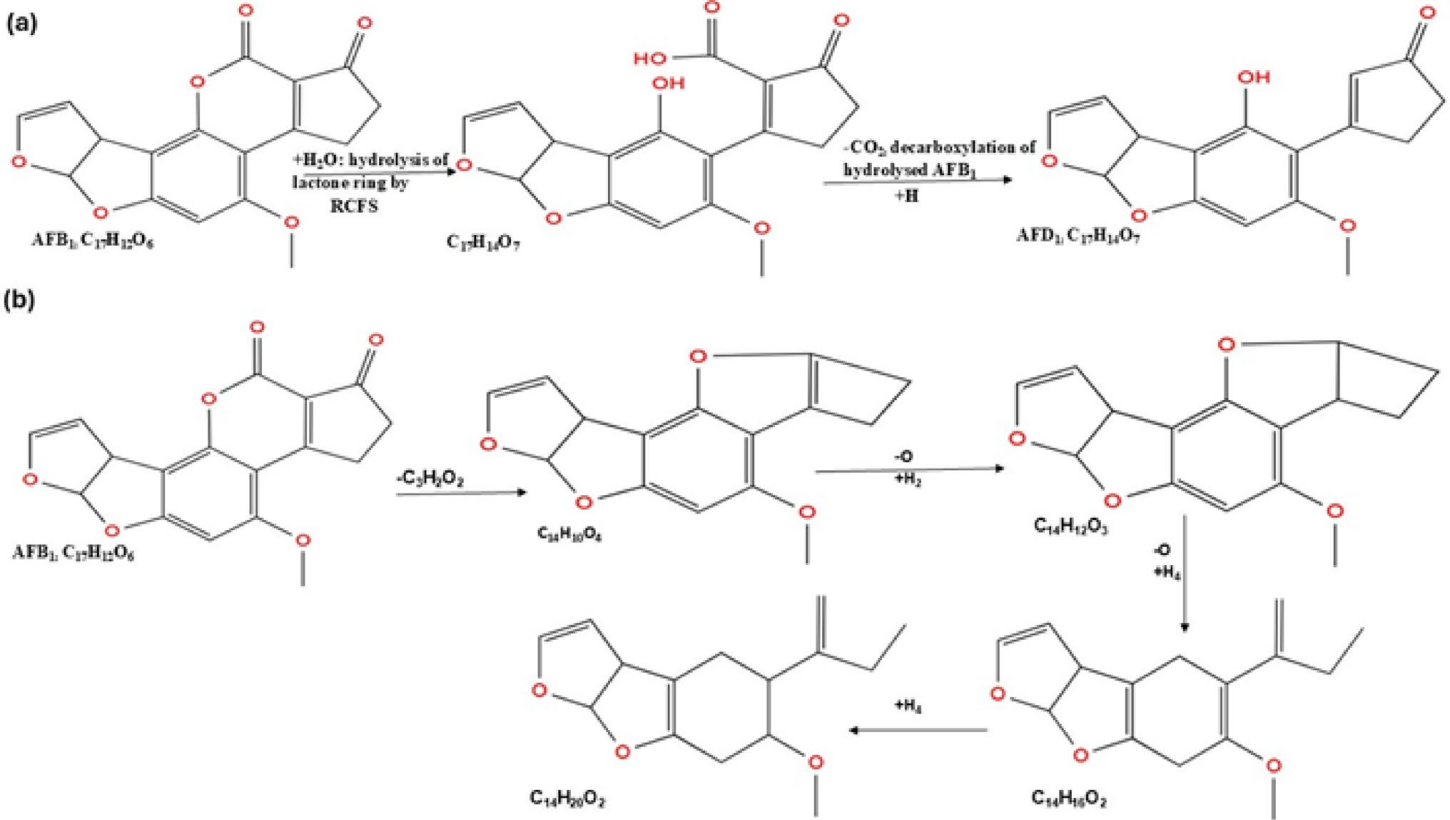
Hydrolysis pathways of aflatoxin B_1_ biodegradation; **a** hydrolysis of AFB_1_ by *R. opacus*, to form less toxic AFD_1_ compound, and **b** hydrolysis of AFB_1_ lactone ring by *Tetragenococcus halophilus* CGMCC 3792

The hydrolysis of the terminal furan ring formed C_11_H_10_O_4_ isomers, followed by epoxidation and rupture of the ester and the ether bonds via _hydrogen_ addition, generating a metabolite with m/z 217.2, and one less C_6_H_2_O_2_ molecule than AFB_1_. The last pathway is the hydrolysis of the lactone ring, resulting in the loss of the methoxy group to the methyl on the benzene ring. The reaction forms a metabolite with m/z 286.3 (C_15_H_12_O_5_), opens the cyclopentenone ring, and acts on the hydroxy to form a m/z 256.1 metabolite (C_14_H_10_O_4_) (Zhang et al. 2021). *Rhodococcus opacus* PD630 cell-free supernatant (RCFS), when treated with AFB_1_, catalyses AFB_1_ to form β-keto acid structure, hydrolyse the lactone, and then decarboxylate the opened lactone, resulting in the formation of AFD_1_ (Fig. 3a). The products retain the furan moiety structure but lose the lactone carbonyl and cyclopentenone ring of AFB_1_ (Zhang et al. 2025).

Li et al. (2018b) described the degradation pathway of aflatoxin, detailing that hydrolysis of the lactone ring led to the loss of one methyl and two carbon monoxides, resulting in a metabolite with a m/z of 243.06 (C_14_H_10_O_4_), followed by a double bond break and hydroxyl group loss, forming a metabolite with a m/z of 229.09 (C_14_H_12_O_3_) (Fig. 3b). The loss of CO opens the benzene ring and ruptures the ether bond, forming a metabolite with a m/z 217.12 (C_14_H_16_O_2_). An extra reaction occurs at the benzene ring, producing a compound with a m/z 221.15 (C_14_H_20_O_2_). The C8-C9 bond is another important ring contributing to aflatoxin toxicity. Wang et al. (2022) reported that hydrolysis of AFB_1_ at this bond results in the formation of AFB_2a_, a less toxic compound compared to AFB_1_.

#### Hydroxylation

Hydroxylation involves the introduction of the hydroxyl group (-OH) to the cyclopentenone ring of the coumarin of aflatoxin. The process is often facilitated by microbial enzymes, which enhance the polarity of the toxins and diminish their toxicity (Li et al. 2020). A study showed that a recombinant type B peroxidase (Rh DypB) could efficiently convert AFB_1_ into the less toxic AFQ_1_. Rh-DypB has proven effective in catalysing the hydroxylation of AFB_1_ to produce non-toxic metabolites, requiring only low enzyme and hydrogen peroxide dosages (Loi et al., 2020).

**Fig. 4:**
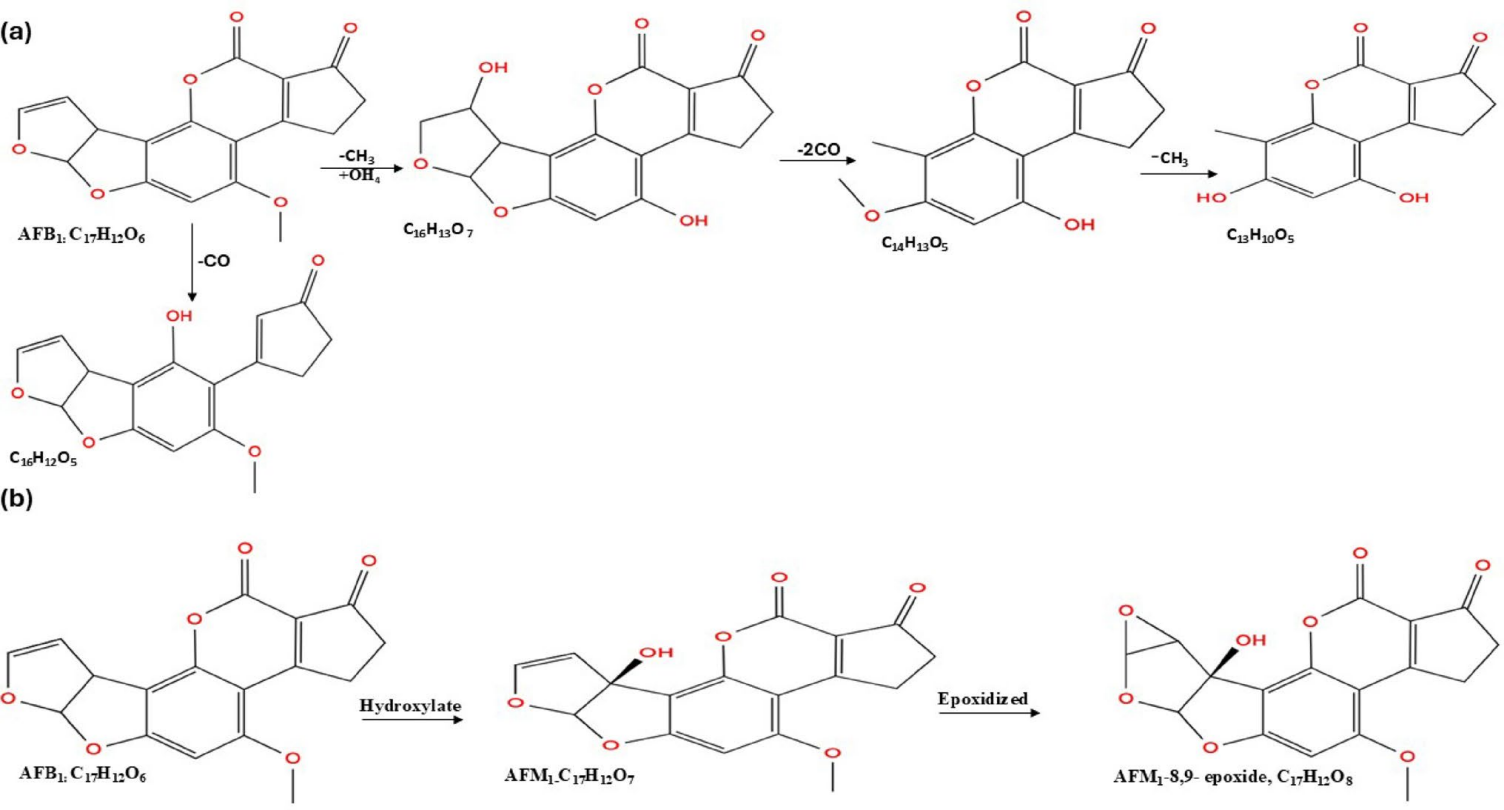
Hydroxylation of aflatoxin B_1_ by **a** *Bacillus* sp. and **b** *R. opacus* into AFM_1_, and then epoxidised into AFM_1_−8,9-epoxide

Extracellular proteins from *Bacillus* strains transforms AFB_1_ through the addition of hydroxy group to the double bond on the left side of the furan ring, yielding a metabolite with molecular formula C_16_H_13_O_7_ (Fig. 4a). The replacement of the AFB_1_ methoxy group on the benzene with the hydroxyl group results in the loss of two carbon monoxides in the furan ring, yielding the metabolite C_14_H_13_O_4_, which subsequently transforms into C_13_H_10_O_5_. Furthermore, removal of the carbon monoxide from AFB_1_ and break down of the ester bond leads to production of a metabolite with molecular formula C_16_H_12_O_5_. This leads to the loss of AFB_1_ fluorescence and mutagenicity activity (Wang et al. 2021).

A degradation product with a m/z 361.09 (C_18_H_16_O_8_) was reported as the downstream metabolite of AFB_1_ through the addition of hydroxyl and methoxyl groups on the double bond of the furan ring (Li et al. 2018b). Another degradation pathway employed by *R. opacus* to detoxify AFB_1_ is hydroxylation, which is formed by the addition of a hydroxyl and methoxy group to the furan ring to form AFM_1,_ which has CH_4_O_2_ molecules than AFB_1,_ as indicated in Fig. 4b. The AFM_1_ has a hydroxyl group attached to its spatial molecular structure, which conjugates with glucuronic acid. This glucuronidation results in a significantly reduced toxicity, estimated at approximately 10% relative to AFB_1_. The AFM_1_ is epoxidised to form a less toxic AFM_1_−8,9-epoxide (Zhang et al. 2025).

Other aflatoxin reductive degradation mechanisms include hydrogenation, decarboxylation, demethoxylation, and demethylation; these are briefly detailed in the following sections.

Demethoxylation of AFB_1_ involves the removal of the methoxy group and its replacement with a hydroxyl group (Li et al. 2020). It has been observed that the demethoxylated products are stabilised by the aromaticity of the benzene ring, as the methoxy group in the C11 position of AFB_1_ plays a role in biodegradation (Arimboor 2024). Li et al. (2018) reported methoxy bond rupture resulting in fewer C_2_O_2_ molecules in the AFB_1_ structure.

Demethylation of AFB_1_ entails the removal of the methyl group (CH_3_), resulting in a metabolite with the chemical formula C_16_H_12_O_5_, as identified by Wang Huang et al. (2021), which is involved in ester bond fracture due to the absence of a methyl group in the benzene ring side chain. Another study showed that the AFB_1_ degradation pathway was formed by the continuous loss of carbon monoxide (CO), with the loss of methyl and methanol mainly occurring on the methoxybenzene located on the side chain, resulting in C_13_H_12_O_2_ with a m/z of 201.13 (Li et al. 2023).

Decarboxylation is an organic reaction that involves the loss of CO. A decarbonylated degradation product was detected by Das et al. (2014). AFB_1_ was degraded by *P. ostreatus* (GHBBF10) to decarbonylated products with m/z 286.2, 283.8 and O-dealkylated (m/z 270) (Das et al. 2014).

**Fig. 5:**
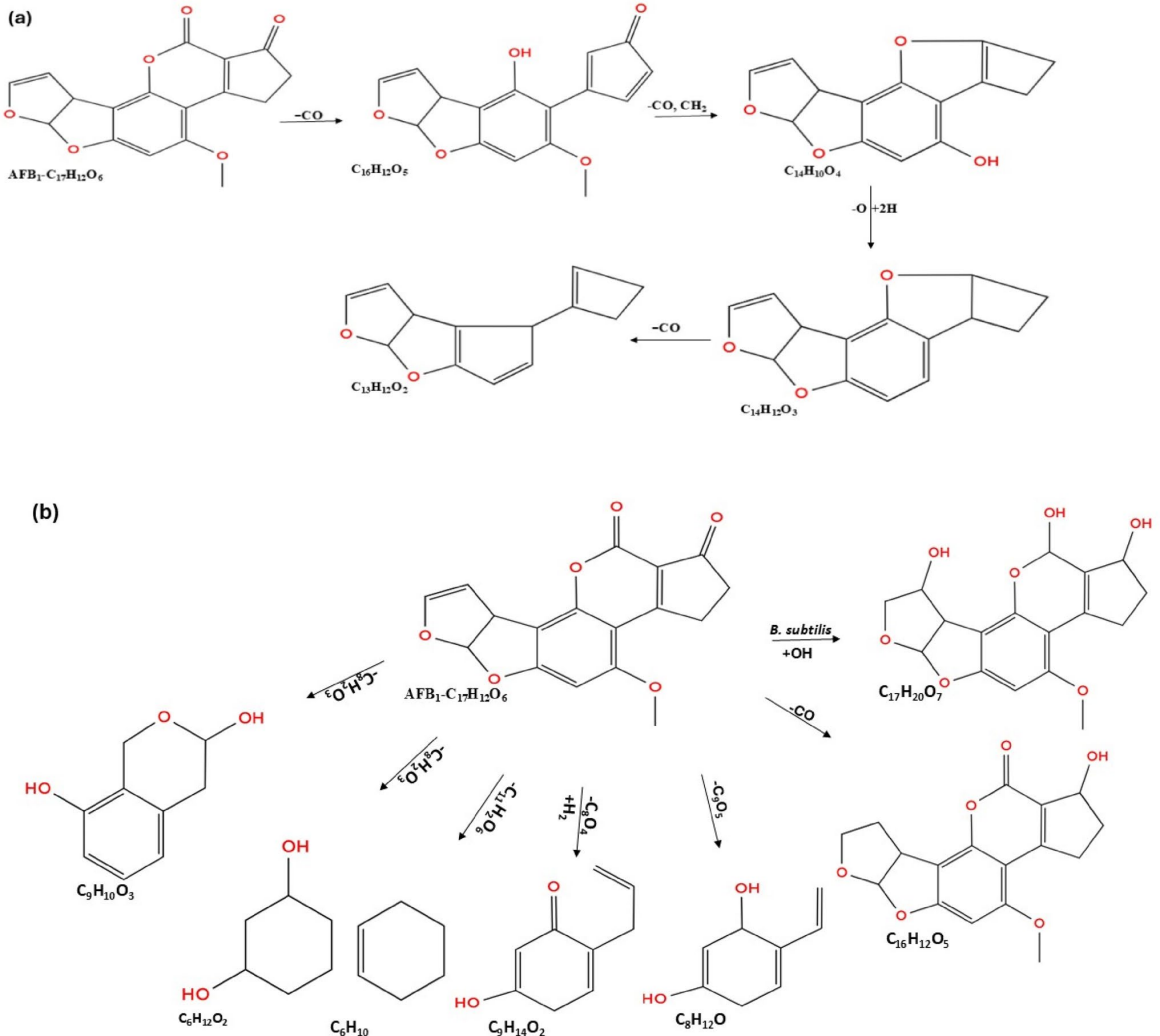
Reductive pathways of mycotoxins. **a** Demethylation of AFB_1_ by *Tetragenococcus halophilus* and **b** decarboxylation, hydrogenation, dehydrogenation, and hydroxylation of AFB_1_ by *B. subtilis*

Hydrogenation reaction changes the structure of a molecule through the addition of hydrogen (H_2_) to an alkene (a double bond). AFB_1_ degradation occurs via direct addition of hydrogen to the cyclopentenone ring at position 14 and the coumarin moiety ring at positions 10, 11, and 15. These enzymatic modification reactions thus disrupt the furan ring at the 8–9 double bond, leading to the formation of the new compounds, C_12_H_14_O_4_ and C_5_H_12_N_2_O_2_. Secondary degradation removes the -CO moiety from the primary degradation products, leading to the formation of less toxic compounds, C_4_H_12_N_2_O and C_10_H_14_O_2_ (Tang et al. 2024). On the other hand, *B. subtilis* CFE transforms AFB_1_ into a less toxic compound through numerous reductive reactions: hydrogenation, dehydrogenation, and hydroxylation, generating products with chemical formulas and m/z values of C_17_H_20_O_7_ (336.12), C_9_H_10_O_3_ (166.06), C_9_H_14_O_3_ (154.10), C_8_H_12_O (140.08), C_6_H_12_O_2_ (116.08), and C_6_H_10_ (82.08) as indicated in Fig. 5b. The final degradation products from this series of reductive reactions (C_9_H_10_O_3,_ C_8_H_12_O, C_6_H_12_O_2_, and C_6_H_10_) confirm the sequence of redox reactions where the furofuran ring was altered or completely divided, and the lactone moiety was lost (Suresh et al. 2020).

### Ochratoxin a degradation reaction mechanisms and pathways

Mycotoxin degradation pathways in fungi are similar to those in bacteria, but some genera, like *Aspergillus*, show unique characteristics, including rapid degradation rates and the generation of unknown compounds (Xiong et al. 2021; Khalil et al. 2021). Metabolic pathways for OTA degradation include hydrolysis, hydroxylation, lactone ring opening and dechlorination. Although the primary pathways in OTA degradation involve hydrolysis (Zou et al. 2022), major OTA degradation products include OTβ from the dechlorination of the isocoumarin ring, OP-OTA from the lactone ring opening, lactone-opened OTA, lactone-opened OTα, ochratoxin hydroquinone (OTHQ) from hydroxylation and OTα and L-β-phenylalanine from cleaving of the peptidic bond (Haq et al. 2016; Wang Lan et al., 2022). OTα consistently remains the major metabolite formed by OTA hydrolysis in humans, animals, and microorganisms (Wu et al. 2011).

**Fig. 6:**
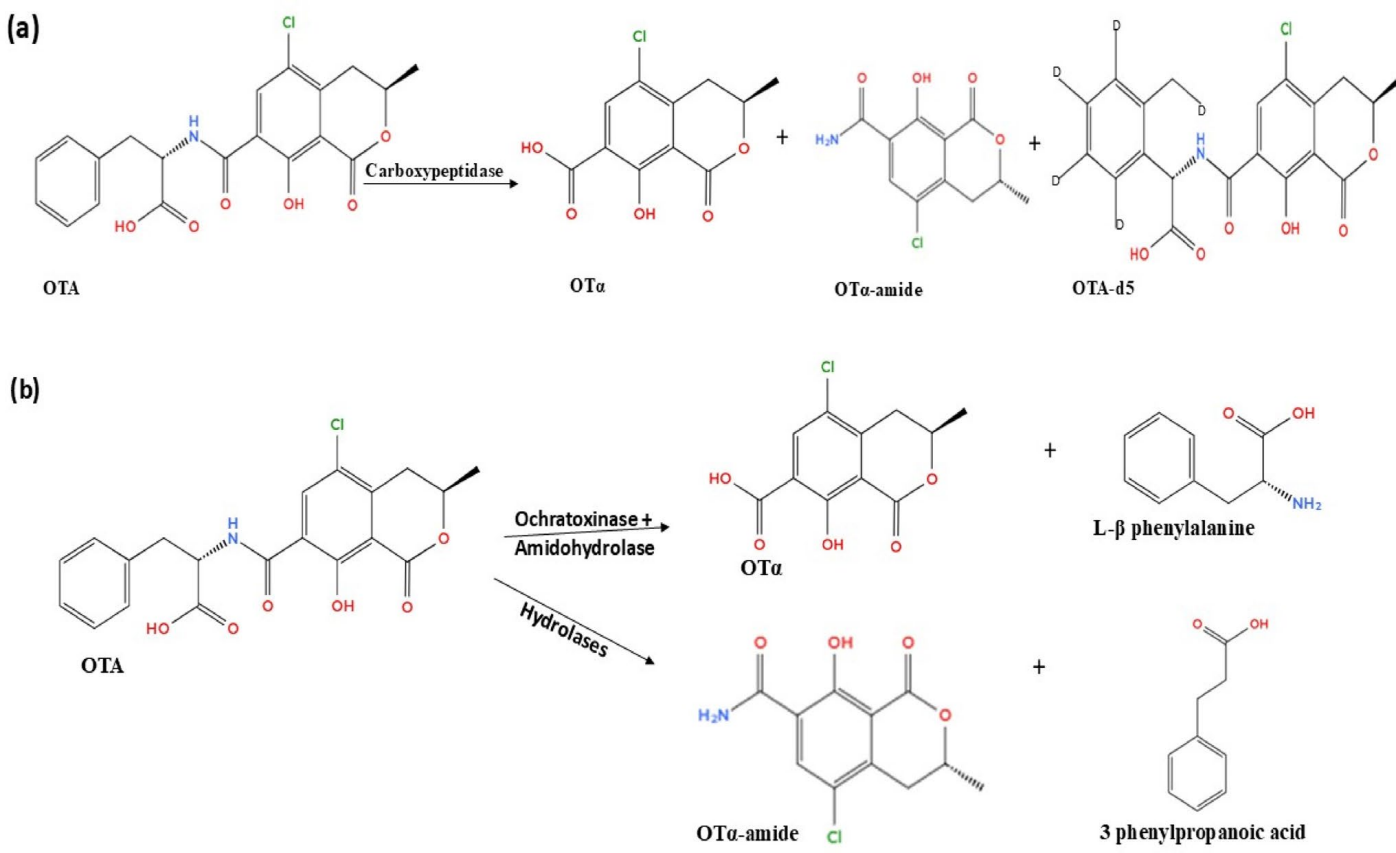
Degradation mechanisms of OTA: **a** hydrolysis by carboxypeptidase and **b** by other hydrolases, ochratoxinase and amidohydrolase

Hydrolysis is one of the major pathways for OTA degradation, whereby this occurs via hydrolysis of the amide bond that links the L-β-phenylalanine molecule to the OTα moiety (Abrunhosa et al. 2010), resulting in the formation of phenylalanine and OTα. In a study by Luo et al. (2022), a recombinant amidohydrolase (ADH3) from *Bacillus* sp. CW117 (1.2 µg/kg) detoxified 50 ng/kg OTA in 90 s; the enzyme catalysed the hydrolysis of the amide bond of OTA, generating OTα. A carboxypeptidase from *Acinetobacter pittii* A19 was identified as one of the contributors to OTA degradation. Recombinant carboxypeptidase (DacC) hydrolysed OTA into non-toxic OTα (Yang et al. 2024). OTA was also hydrolysed by a recombinant carboxypeptidase to the non-toxic metabolites; OTα (239.60 m/z), OTα-amide (256.40 m/z), and ochratoxin A-d5 (408.53 m/z) (Azam et al. 2019) as presented in Fig. 6.

Two distinct OTA biodegradation pathways are employed by *Trichoderma koningii* AUMC11521 enzymes, involving the hydrolysis of the amide bond by ochratoxinase with amido-hydrolase activity, generating non-toxic OTα and L-β-phenylalanine, and hydrolases producing OTα-amide and 3-phenylpropanoic acid. The enzymes cleave the amide bond resulting in the breakdown of OTA (Ismaiel et al. 2023).

**Fig. 7:**
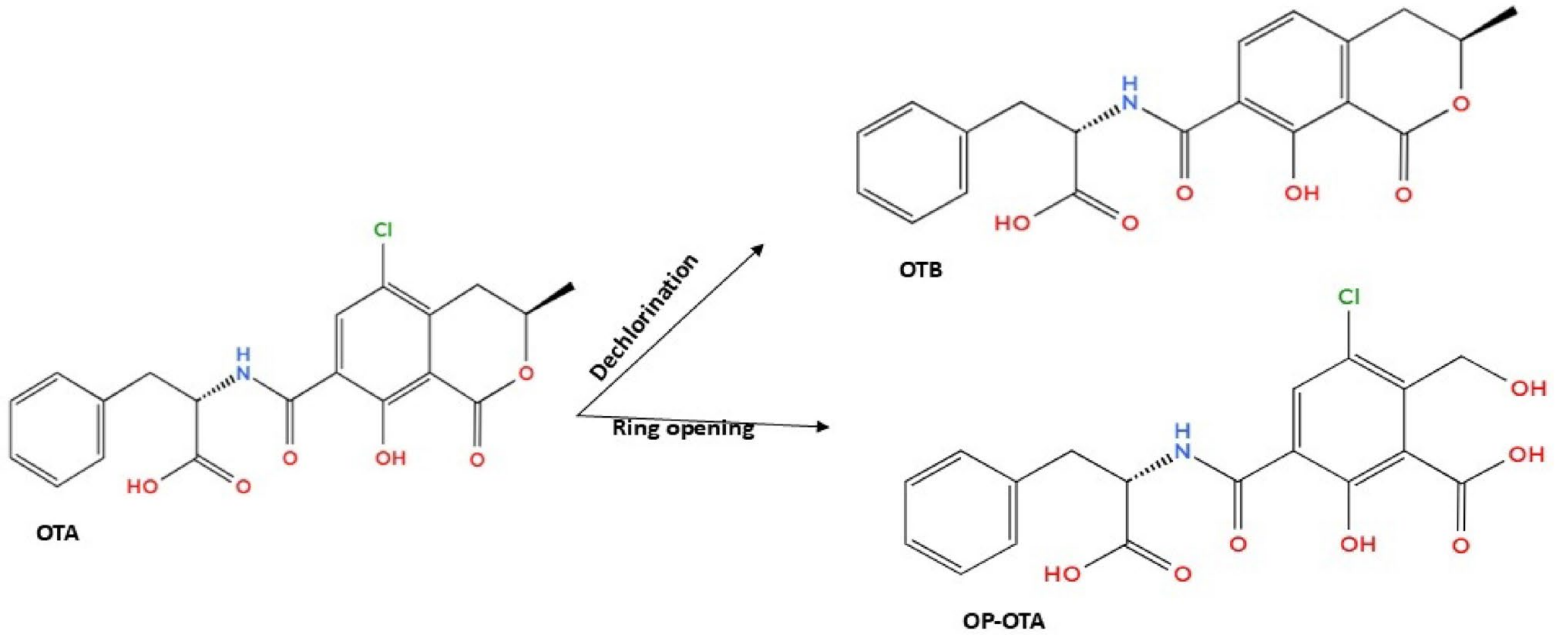
Dechlorination and ring opening of OTA by microbiota

Additional OTA biodegradation mechanisms include its dechlorination into OTB and opening of the lactone ring to generate OP-OTA (Fig. 7). Dechlorination and ring opening of OTA were reported by Camel et al. (2012) after incubating OTA with the human intestinal microbiota; the degraded metabolites identified include OTB and OP-OTA. OTA is metabolised through the hydrolysis of peptic bond to form OTα, followed by dechlorination of the isocoumarin moiety leading to the formation of OTB, and the opening of the lactone ring to form OP-OTA. OTα and OTB are less toxic than the OTA. Dechlorination of OTA can generate reactive aryl radicals, leading to the production of purine nucleotide adducts. Disruption of the natural toxicokinetics of OTA by inhibiting its cellular absorption, represents a potential strategy for preventing cellular build and mitigating its harmful effect in the human body (Kőszegi and Poór 2016).

OTA by-product, L-β-phenylalanine, can be metabolised via the branched-chain amino acid pathway by enzymes such as glutamine synthetase, which catalyses the synthesis of glutamine from glutamate and ammonia, and branched-chain and aromatic aminotransferase that catalyse the transamination of branched-chain amino acids (Jelku and Sangma, 2024; Zhang et al. 2018).

## Applications and industrial development of enzymes-based mycotoxin technology in food and feed

Application of enzymes to food matrices for mycotoxin degradation is an effective strategy for biodegradation. In food and feed industries, enzymes are applied as either processing aids or food additives added directly to the food matrices such as corn bran, maize by-products, and wheat flour (Bi et al. 2018; Kupski et al. 2018) or immobilized on solid supports to enhance their stability and reusability in food (sugar, fish, and wine) processing (Sirisha et al. 2016). Application of immobilised pectinase could hydrolyse the pectin and thus produce the fruit juices with no pectin residues (Hiteshi et al. 2013). Commercial laccase significantly improved the bread making performance of oat flour and textural quality of bread, and lowering crumb hardness (Renzetti et al. 2010). Immobilised Mnp effectively removed 42.7% bitterness from fruit juices, reducing the colour and turbidity by 36.3%. After six reusability cycles, the immobilised retained 60–63% of its initial activity in oxidising phenolic compound in apple and orange juice (Bilal et al. 2016). In recent years, the number of industrial uses of enzymes has increased dramatically due to the developments in protein engineering technology, which allow the integration of structure-based rational design with computational techniques (Choi et al. 2015). These approaches give room for the enzymes to be immobilised and used in a controlled manner, extending the shelf life of the food and making the enzymes’ stability more efficient than when they are directly incorporated (Motta et al. 2023).

## Advantages and disadvantages of mycotoxin biodegradation approaches

Although most of the chemical methods achieved 100% degradation, they are incompatible with organic farming and the current global context of climate change due to issues such as the presence of chemical residues in the produce, which contravene organic farming regulations (Peng et al. 2018; Kos et al. 2023). In contrast, biocontrol offers a promising, eco-friendly mycotoxin degradation method, compatible with organic farming principles (Kos et al. 2023; Mahmoud et al. 2025; Rahman et al. 2025).

Non-biological control for mycotoxin degradation may result in several food quality issues, such as alteration of sensory properties, formation of harmful by-products, lipid oxidation, and loss of the functional qualities of macromolecules in the produce (Esua et al., 2021; Cai et al. 2025). However, Saremnezhad et al. (2021) propose that improving these procedures may reduce their limitations. Biocontrol methods show little or no negative impacts on food quality.

The effectiveness of biocontrol methods is supported by their correspondence with advanced microbial enzymatic strategies outlined in recent research. An illustrative case is the work of Shaji et al. (2024), which examines microbial consortia and enzymatic mechanisms for plastic degradation, reflecting the principle of employing specific microbes or their enzymes to effectively target and decompose mycotoxins without generating harmful residues. The challenges of maintaining efficacy in complex environments, as highlighted for the non-biological methods, can be effectively addressed through the immobilisation techniques reviewed by Saravanan et al. (2023). Immobilising mycotoxin-degrading microbes or enzymes on supportive matrices enhances their stability and functional longevity in food systems, providing a durable and efficient solution that preserves nutritional quality. This approach, in conjunction with the precise detection methods outlined by Saravanan et al. (2021), establishes a robust framework for remediating contaminations and monitoring food safety while maintaining the organic integrity and quality of the produce.

## Challenges associated with the biological control of mycotoxins

Biocontrol agents offer a wide range of benefits across various food industries, including, among others, their ability to combat mycotoxigenic fungi and neutralise their mycotoxins. However, their mycotoxin degradation capability is primarily strain- and culture medium-dependent (Taheur et al. 2017). LAB and *Bacillus* spp. isolated from wheat grains were tested against zearalenone (ZEA) and deoxynivalenol (DON), out of 212 investigated strains, only 42 strains, i.e., *Bacillus licheniformis (*19 strains), *B. megaterium* (13), and *Levilactobacillus brevis* (10) showed the ability to detoxify 90% ZEA (Mischler et al. 2024). The unique mycotoxin-degradation capabilities of each microbial strain necessitate individual testing before use, making the approach challenging. Biodegradation of mycotoxins also has the disadvantage of being time-consuming (Benites et al. 2017).

Biocontrol agents, such as enzymes, are often sensitive to temperature, pH, humidity and water activity fluctuations in the food matrix. For example, carboxypeptidases have an ideal temperature of 30 °C or higher; therefore, applying them to food matrices below this temperature may reduce their activity. The biodegradation of OTA by carboxypeptidase A from *M. esteraromaticum* ASAG1016 was temperature dependent. At 37 °C to 45 °C, the degradation rates were higher, resulting in 90% degradation and above, whereas at 60 °C, the degradation rates decreased significantly, because the high temperature denatures and inactivates the enzyme (Jia et al. 2025).

Similarly, changes in pH affect the degradation efficiency of the biocontrol agents as pH influences hydrophobic and electrostatic interactions between the mycotoxin and the microbial cell wall (Adebiyi et al. 2020). Dehulling Bambara groundnut with fermentation to produce *dawadawa* effectively reduced mycotoxins. The longer duration of the fermentation process and increase in temperature and pH also influence mycotoxin degradation (Adebiyi et al. 2020). Decomposition of AFB_1_, OTA and ZEA by *L. acidophilus* and *L. rhamnosus* was most efficient at acidic conditions, as this led to an increase in fermentation time, reducing mycotoxins in dough samples, while the mycotoxin degradation capacity decreased at neutral to alkaline conditions (Pourmohammadi et al. 2022).

Another challenge with the biodegradation of mycotoxins is that it can produce unknown or unanticipated by-products, some of which may be harmful. These by-products require toxicity tests, and their interactions with food components need to be evaluated before being applied to food products (Pankaja et al. 2018; Xiong et al. 2021).

Probiotic strains, including those with mycotoxin-degrading capabilities, can interact with food pathogens present in food through several mechanisms and produce antagonistic metabolites, such as hydrogen peroxide and organic acids (Khaneghah et al. 2020).

## Strategies for improving the efficiency of biological mycotoxin detoxification methods

The process of screening and selecting microbial strains or consortia focuses on identifying the most effective microbial strains or consortia for biotransformation activities. Most strains are chosen based on their capacity to generate extracellular enzymes, their use as probiotics, their antimicrobial properties, cell wall hydrophobicity, and their resilience under challenging environmental conditions (Zhu et al. 2017). These organisms can also improve the soil health which have significant positive environmental impacts (Nawaz et al. 2025). A microbial consortium can leverage the advantages of each strain to enhance stability and efficiently degrade contaminants (Zhang and Zhang 2022). The biodegradation of mycotoxins can utilise genetically engineered microbes to improve the efficiency of the degradation process. Other strategies for improving the efficiency of biological mycotoxin detoxification are discussed below.

### Enzyme discovery and immobilisation

Proper identification of enzymes involved in mycotoxin degradation and the use of various substrates is important. As most of the crystal structures of mycotoxin-degrading enzymes are still unknown, the use of structural biology to identify important protein residues can help researchers to rationally design these enzymes to improve their stability and efficiency (Shi et al. 2023).

The self-assembling protein scaffolds can then be used to immobilise the enzymes, improving their stability, activity, and reusability. The chitosan-immobilised recombinant laccase exhibited higher AFB1/ZEN degradation activity than free laccase. Immobilised enzymes demonstrated improved efficiency and reusability, placing them as a favourable option for enhanced biodegradation performance (Gao et al. 2024). Microencapsulation and nanotechnology will aid the stability and activity of the enzymes. Immobilisation of bacteria, especially gut microbes, shields the organisms from environmental stress, ensuring their stability and stability throughout digestion (Jelku and Sangma 2024). It is envisaged therefore, that these same benefits will be extended to enzymes. Enzymes can also be immobilised and introduced to degradation medium or solid support materials that enable the transfer of degrading chemicals into it (Karishma et al. 2023).

### Optimisation of environmental conditions in the food matrix

Optimisation of environmental conditions, such as temperatures and pH, will improve the efficiency of the biodegradation process. It is crucial to evaluate the degradation process at the same temperature and pH level as the food matrix temperature. In vivo analysis of the biocontrol agent must be performed to determine the suitable conditions for the activity of microorganisms and enzymes in food matrices. Abrunhosa et al. (2014) propose inoculating food matrices with biocontrol agents to test their efficacy in natural environments. Also, replacing the residues at the region responsible for enzyme thermostability can improve the resistance to high temperatures.

Ultimately, accurate identification and toxicity assessments are crucial for the validation and acceptability of biodegradation, as well as for the structural elucidation of the degraded by-products. Analysis of degraded toxin products conducted by Iram et al. (2015) utilising LC/MS and PDA plus detectors indicated that the toxicity of AFB_1_ and AFB_2_ degradation by-products was markedly reduced compared to that of the parent molecule. Furthermore, conducting tests on the by-products of biological mycotoxin degradation, including mutagenicity tests, cell viability assessments, rat toxicity evaluations, and cell apoptosis assays, will enhance the credibility of biological degradation (Shi et al. 2023).

## Conclusion and future perspectives

Biodegradation has evolved as an effective and increasingly feasible approach for reducing mycotoxin contamination in food and feed systems, suitably positioned to become a next generation mycotoxin control strategy. It has notably transitioned from just a conceptual detoxification method to an in-depth credible and transformative intervention. Various microbial species and their enzymes, have shown the ability to degrade significant mycotoxins, including aflatoxins and OTA, without generating toxic intermediates. Initial research concentrated on the detoxification of individual mycotoxins through enzymatic systems, however, the field now stands at a critical inflection point, where mechanistic and technological maturity must converge. Such convergence will signify a shift from passive management of mycotoxin contamination to active detoxification. Another significant limitation of existing research is the predominant focus on and reliance on in vitro models. The urgent requirement for additional in vivo validation within real food and feed matrices has become apparent. The in vivo evaluations represent a step closer to translation into real-world applications, where efficacy and performance is dependent on the food or feed matrix effects, enzyme stability and interactions with natural microbiota, as well as processing conditions.

Evidence accumulated over the years demonstrated that microbial enzymes, particularly hydrolases such as lactonases, carboxypeptidases, and proteases, consistently achieve complete detoxification of mycotoxins. The risk associated with the oxidative transformation of the aflatoxin furan ring is significant, as it can produce reactive epoxide intermediates that can traverse nuclear membranes and create DNA adducts associated with AFB_1_ carcinogenicity. The oxidative pathways are therefore, incompatible with long-term safety objectives. These findings underscore a pivotal paradigm shift; effective mycotoxin control must prioritise irreversible molecular detoxification over partial transformation. Additional research is necessary to clarify the biochemical pathways and catalytic mechanisms related to the detoxification of aflatoxin and OTA, and to determine the physicochemical conditions that optimise enzyme efficiency for effective mycotoxin biodegradation. The thorough evaluation of biodegradation by-products is crucial for ensuring their safety for consumers, animals, and the environment. Toxicological profiling of biodegradation by-products must be addressed to confirm their suitability for consumers, livestock, and the environment, thereby strengthening standardisation and regulatory confidence for policymaking and public acceptance.

Future research must concentrate on the standardisation, optimisation, implementation, and comprehensive industrial deployment of highly active, purified microbial enzymes that have proven efficacy against aflatoxins and OTA. Enzymatic detoxification by itself, cannot function as a comprehensive solution, but will require its combination with preventive and non-thermal technologies. Examples of the preventive strategies such as good agricultural practices (GAPs), enhanced postharvest handling, and efficient sorting will ensure comprehensive management throughout the food and feed value chain. Cold plasma is a non-thermal technology that inhibits fungal growth and suppress aflatoxin biosynthesis, as well as enhance enzymatic accessibility, features that position it as a sustainable, environmentally friendly alternative to chemical detoxification methods.

This review revealed notable knowledge gaps, particularly the lack of reliable assessments of biodegradation effectiveness in real food and feed matrices, insufficient evaluation of the safety of degradation by-products, and limited evidence supporting feasibility for large-scale applications. These gaps will be filled through next generation studies that holistically focuses on toxicological validation, in vivo tests, multi-modal detoxification systems and regulatory and policy frameworks. Comprehensive profiling of mycotoxin biodegradation by-products will be achieved through the use of advanced methods such as metabolomics, protein engineering, and probably even artificial intelligence-guided enzyme optimization approaches. This will be a much-needed transformation, especially for high-risk commodities such as spices which presents quantifiable public health concern, especially for vulnerable individuals, such as those with hepatitis B. Probabilistic modelling already demonstrates a disproportionate mycotoxin exposure and higher cancer risk in these populations. Biodegradation stands to inform or redefine food safety, with enzymatic biodegradation holding the potential to emerge as an industrial standard for mycotoxin decontamination. Ultimately, enzymatic mycotoxin degradation represents a pathway towards attainment of sustainable public health protection and enhanced food security.

## Data Availability

No datasets were generated or analysed for this article.
